# What Factors Impact upon a Woman’s Decision to Undertake Genetic Cancer Testing?

**DOI:** 10.3389/fonc.2013.00325

**Published:** 2014-01-06

**Authors:** Julie A. Quinlivan, Zain Battikhi, Rodney W. Petersen

**Affiliations:** ^1^North Metropolitan Health Service, Western Australian Department of Health, Perth, WA, Australia; ^2^Institute of Health Research, University of Notre Dame Australia, Fremantle, WA, Australia; ^3^Obstetrics and Gynaecology, University of Adelaide, Adelaide, SA, Australia

**Keywords:** breast cancer, genetic cancer screening, pregnancy, discrimination, ethics, education

## Abstract

**Introduction:** The advent of human genome project has lead to genetic tests that identify high-risk states for certain cancers. Many are privately marketed on the Internet. Despite the availability of tests, limited data has evaluated factors that lead to test uptake. The aim of the present study was to explore the attitudes of a cohort of new mothers toward uptake of a genetic cancer test with a 50% predictive value of cancer.

**Methods:** A cross-sectional survey was undertaken. The project targeted women who had recently given birth at an Australian tertiary referral hospital. Women were asked about a theoretical blood test that detected an increased risk for the development of cancer. Attitudes and knowledge questionnaires were completed.

**Results:** Of 232 consecutive women approached, 32 declined, giving a response rate of 86.2%. Only 63 (31.5%) women stated they would have the test. Absence of religious belief, higher level of education, better knowledge of terms used in genetics, an absence of concern over emotional, employment, and insurance discrimination, and previous acceptance of Down syndrome screening in pregnancy were each associated with significantly higher rate of test uptake in univariate analysis (all *p* < 0.03). In multivariate analysis, a lack of concern over discrimination and a history of having accepted Down syndrome screening in the previous pregnancy remained significantly associated with test uptake (all *p* < 0.0001).

**Conclusion:** Concern over discrimination and having made a prior decision to have genetic testing were the principal factors associated with decision-making.

## Introduction

The advent of human genome project has lead to genetic tests that identify high-risk states for certain cancers. Commercial companies now market the availability of these tests on the Internet. However consumer agencies and medical staff are concerned this will lead to medicine for the rich and inadequate pre-test counseling (1–5).

Whilst healthcare providers offer recommendations and guidance about genetic cancer testing to screen appropriate patient groups, ultimately, the onus is left on patients and their families to decide whether or not to uptake testing.

The published literature has identified several factors that influence this decision. One study compared the impact of demographic variables, medical history, and psychological factors on uptake of genetic testing for hereditary colorectal cancer (1). An association was found between increased test uptake and those candidates with increased risk perception, greater perceived confidence in their ability to cope with unfavorable genetic information, more frequent cancer thoughts, and those having undergone previous screening (colonoscopy examination) (1). Similar findings have been reported in studies investigating the association between psychological factors and cancer screening behaviors (2–6).

Other documented motivating factors influencing decisions to uptake genetic testing include a desire to seek information on personal and family (especially offspring’s) risk of disease, to detect cancer at an early stage, and to contribute to research in the field of genetic testing (7). Conversely, concerns over loss of health insurance and the potential psychological consequences of testing were quoted in other studies as reasons for declining test uptake (8, 9). Limited data has evaluated the impact of knowledge of genetics and attitudes toward genetic-based discrimination in combination with actual testing behaviors.

The aim of this study was to explore the relative impact of demographic variables, level of knowledge, attitudes on potential discrimination, and previous genetic testing behavior on a woman’s decision as to whether she would accept or decline a genetic cancer test that could predict the onset of cancer within the next 5 years.

## Materials and Methods

A cross-sectional survey was undertaken. The study had the approval of the Institutional Human Research and Ethics Committee. Individual informed written consent was obtained from each participant. The project targeted women who had recently given birth at an Australian tertiary referral hospital. Non-English speaking women were specifically included in the study by utilizing qualified medical interpreters and research assistants who were fluent in several languages.

The recruitment of subjects took place in the postnatal wards of the hospital. All participants had previously had to make a personal decision about accepting or declining genetic testing in relation to their pregnancy in respect to Down syndrome screening or diagnostic testing, and on behalf of their baby with respect to the newborn screen.

### Questionnaire design

Women were asked a specific question about a theoretical blood test that could detect an increased risk for the development of cancer. The question was piloted on a focus group before being finalized. The starting point of the question assumed a woman had undergone a pedigree analysis. The woman was then asked the question:

“Imagine that there was a blood test that could tell you that there is a 50% chance (that is 1 in 2 chance) that you will get a specific cancer within 5 years time. Would you take the test?”

An attitudes questionnaire was then completed that covered women’s attitudes toward potential discrimination in relation to genetic testing. Women were asked whether they thought a positive genetic result would be associated with

- an effect on emotional and psychological development
- an effect on obtaining insurance
- an effect on employment.

Participants also completed a knowledge questionnaire that was designed to evaluate the knowledge of women in respect to key terms used in counseling for genetic testing. The details of this questionnaire have been previously described (10). The score for the knowledge questionnaire ranged from a minimum of 0 to a maximum of 15 with median score of 4.

The final part of the questionnaire covered demographic factors, family history of genetic disease (apart from cancer), and cancer. A readability formula, the SMOG index, was applied to the wording of the questionnaire (11). This ensured that the readability of the questions was in accordance with the published literacy figures for the Australian population. The resulting SMOG index of the questionnaire was 8. This meant that important concepts in the questionnaire would be understood by 90% of readers who had completed grade 8.

### Sample size and statistical analysis

The primary hypothesis was that fear of discrimination may deter women from seeking out tests that might detect an increased risk of cancer. It was predicted that a fear of discrimination might reduce test uptake from 50 to 25%. Given a two-sided α error of 0.05 and β error of 0.80, 65 women would be required as a minimum in each arm of the study. We predicted that 35% of women may worry about discrimination, and therefore predicted that at least 186 women would need to be enrolled in the study. We therefore aimed for a population target of 200.

Data collected were analyzed using Epi Info, Centre for Disease Control. Demographics of the study cohort were compared against the broader demographics of the Victorian maternal population in respect to age, marital status, primary language, religion, and parity in order to assess the generalizability of findings in the study cohort to the wider obstetric population (12, 13).

Descriptive statistics were obtained on individual questions. Discrete variables are reported as *N* (%) and were compared using Chi square or Fishers Exact test according to cell size. Continuous variables are reported as mean (standard deviation) or median (interquartile range) and were assessed using Students *t*-test or Mann–Whitney *U* test according to distribution. Results were also compared against the women’s previous decision in relation to Down syndrome testing.

Multivariate analyses of variance was performed with the model including factors found to be significant on univariate analysis at a *p*-value <0.1 The dependent variable was test uptake. A *p*-value of 0.05 was considered significant.

## Results

Of 232 consecutive women approached, 32 declined, giving a response rate of 86.2%. The commonest reasons for the mother declining participation were that they were too busy with their newborn babies or tired.

The demographics of the cohort are summarized in Table 1 which compares the demographic details of the study cohort and to data available for the State of Victoria Australia. Key demographics were similar. However proportionally more women with a religious belief stated it was Islam compared to the breakdown of type of religion in population data for the State, although the total proportion of women with any religious belief was similar. As there were no differences by type of religion for any variable, data on religious belief were pooled for analysis.

**Table 1:** **Demographics**.

Variables	Study population	Victorian data
Age in years	29.1 (5.6)	29.7
Mean (SD)	
Marital status
Married *N* (%)	146 (73%)	75.3%
English as the first language *N* (%)
Yes	117 (58.5)	
No	83 (41.5)	
Ethnic background *N* (%)
Caucasian	86 (43)	
Mixed race	64 (32%)	
Asian	28 (14)	
ATSI	14 (7%)	
Pacific islander	5 (2.5%)	
African	3 (1.5%)	
Religious belief
Yes *N* (%)	149 (74.5%)	74.4%
Level of education *N* (%)
No school or 1^0^ school only	8 (4%)	
Attended part of 2^0^ secondary school	50 (25%)	
Completed secondary school	142 (71%)	
Income of family before tax *N* (%)
<$20,000	36 (18%)	
$20,000–$30,000	32 (16%)	
$30,000–$40,000	43 (21.5%)	
>$40,000	67 (33.5%)	
Not disclosed	22 (11%)	
Parity *N* (%)
1	86 (43%)	41.7%
2 or more	113 (56.5%)	
Did not answer	1 (0.5)	

Only 63 (31.5%) of women stated that they would be prepared to have a blood test that might tell them that they had a 50% risk of acquiring a cancer in the next 5 years.

Table 2 summarizes the impact of demographic variables on the decision for test uptake. Women without a religious belief were significantly more likely to consider test uptake compared to those without (44 vs. 27%, *p* = 0.02). Level of education also significantly influenced the decision for test uptake (*p* = 0.02). Of note, the variables of family history of genetic disease (except cancer) and family history of cancer did not significantly impact upon the decision to have the theoretical test.

**Table 2:** **Impact of demographic factors on decision on test uptake**.

Variables	Accept test (*N* = 63)	Decline test (*N* = 137)	*p*-Value (univariate)	*p*-Value^c^ (multivariate)
Demographics
Age mean (SD)	28.9 (5.2)	29.2 (5.8)	0.77	
Marital status
Married	50 (34%)	96 (66%)	0.19	
*Defacto*	11 (30%)	26 (70%)	
Single	2 (1%)	14 (88%)	
Missing data	0	1	
English first language
Yes	33 (28%)	84 (72%)	0.23	
No	30 (36%)	53 (64%)	
Ethnic background
Caucasian	33 (38%)	53 (62%)	0.18	
Asian	8 (40%)	20 (60%)	
Other^a^	22 (24%)	64 (76%)	
Religion
No religion	22 (44%)	28 (56%)	0.02	0.17
Religion	40 (27%)	109 (73%)	
Subtype of religion
Christian	23 (29%)	56 (71%)		
Islam	14 (32%)	30 (68%)		
Other^b^	3 (12%)	23 (88%)		
Missing data *n* = 1	1	0		
Education
No school or 1^0^ school only	0 (0%)	8 (100%)	0.02	0.13
Attended part of 2^0^ secondary school	11 (22%)	39 (78%)	
Completed secondary school	52 (37%)	90 (63%)	
Income
<$20,000	7 (19%)	29 (81%)	0.09	0.46
$20,000–$30,000	12 (37%)	20 (63%)	
$30,000–$40,000	20 (47%)	23 (53%)	
>$40,000	24 (36%)	43 (64%)	
Missing data	0	22	
Parity
1	27 (31%)	59 (69%)	0.94	
2 or more	36 (32%)	77 (68%)	
Missing data	0	1	
Family history of genetic disease (except cancer)
Yes	8 (36%)	14 (64%)	0.60	
No	55 (31%)	123 (69%)	
Family history of cancer
Yes	18 (43%)	24 (57%)	0.07	0.37
No	45 (28%)	113 (72%)	

^a^Mixed race, Aboriginal and Torres Strait islander, Pacific islander, African.

^b^Jewish, Johovah Witness, Buddhist, Scientology, Fundamental (unclassified), or Special (unclassified).

^c^Multivariate analysis ANOVA included religion, education, income, family history of cancer, knowledge of genetics, emotional disadvantage, insurance disadvantage and employment disadvantage, and previous behavior in relation to Down syndrome testing in the preceding pregnancy.

Table 3 summarizes the influence of knowledge of genetic terminology, attitudes, and previous decision making in respect to Down syndrome testing on the decision to consider testing for cancer risk. Women who stated they would have testing to determine if they were at 50% risk of developing cancer in the next 5 years had significantly higher levels of knowledge of terms used in genetic counseling (score 5 vs. 3, *p* = 0.03). Women who were concerned about possible emotional, employment, or insurance disadvantage as a result of being a carrier were significantly less likely to undertake testing (all *p* < 0.0001). Women who had elected to have screening or diagnostic testing for Down syndrome in their preceding pregnancy were significantly more likely to agree to cancer testing (55 vs. 3%, *p* < 00001).

**Table 3:** **Impact of genetic knowledge, attitudes toward carrier status, and previous decision-making on Down syndrome investigations on test uptake**.

Variables	Accept test (*N* = 63)	Decline test (*N* = 137)	*p*-Value (univariate)	*p*-Value^b^ (multivariate)
Knowledge of genetics^a^
Median (IQR)	5 (3–8.5)	3 (1–8)	0.03	0.19
Attitudes to carrier status
Emotional disadvantage
No	37 (69%)	17 (31%)	<0.0001	<0.0001
Uncertain	15 (22%)	52 (78%)	
Yes	11 (14%)	66 (86%)	
Missing data	0	2	
Insurance disadvantage
No	40 (80%)	10 (20%)	<0.0001	<0.0001
Uncertain	12 (15%)	67 (85%)	
Yes	11 (16%)	59 (84%)	
Missing data		1	
Employment disadvantage
No	42 (52%)	38 (48%)	<0.0001	<0.0001
Uncertain	11 (19%)	48 (81%)	
Yes	10 (17%)	50 (83%)	
Missing data	0	1	
Decision on down syndrome testing
Underwent a test	60 (55%)	48 (45%)	<0.0001	<0.0001
Declined testing	3 (3%)	89 (97%)	

^a^Range 0–15: 0 = low knowledge, no correct answers in test to 15 = high knowledge, all answers correct in test.

^b^Multivariate analysis ANOVA included religion, education, income, family history of cancer, knowledge of genetics, emotional disadvantage, insurance disadvantage and employment disadvantage, and previous behavior in relation to Down syndrome testing in the preceding pregnancy.

The multivariate analysis included all variables significant at a univariate level of *p* < 0.1. A belief that a positive result would be associated with discrimination and a history of having accepted screening for Down syndrome in the previous pregnancy remained significantly associated with the decision on test uptake (all *p* < 0.0001) (Tables 2 and 3).

## Discussion

Various studies over the past couple of decades have investigated the factors impacting upon decisions to undertake genetic testing predictive for cancer as well as other conditions, including sickle cell disease (14), colon cancer (1), bipolar disorder (15). This study is unique, however, in that the focus was shifted from considerations pertaining to the nature of the actual medical condition tested for, to a holistic exploration of the relative impact of multiple psychosocial factors, alongside demographic and behavioral variables, on a woman’s decision to accept or decline a hypothetical genetic test that could predict the onset of cancer within the next 5 years. These factors included family history of genetic disease, family history of cancer, knowledge about terms used in genetic testing counseling, actual behavior in relation to a decision to accept or decline genetic testing of their fetus in an immediate prior pregnancy and attitudes toward potential discrimination. Our study is also unique for the target population cohort studied.

A higher tendency toward test uptake was found in our postnatal women cohort who did not have concerns in relation to emotional, employment, or insurance discrimination and who had better knowledge of terms used in genetic counseling. Moreover, and of greater significance perhaps, was the multivariate analysis that found an independent adverse association between the decision on test uptake and a belief that a positive result would be associated with discrimination; and a favorable association in women with a prior history of having previously accepted screening or diagnostic testing for Down syndrome in their preceding pregnancy. These findings highlight a potentially pertinent limitation to the advent and use of genetic testing to screen for certain disease states; that is, cautious acceptance of these proposed tests and hesitancy toward uptake by a general public who are fearful of the potential professional and personal implications that the test results may yield. In essence, this represents hypothetical fear of genetic-based discrimination.

### Genetic-based discrimination

Many definitions of genetic-based discrimination have been offered by sociocultural and scholarly institutions in response to the increasing availability of genetic testing over the past three decades. Debate in this field has focused on the potential implications of genetic-based discrimination, including risk of actual or feared psychological/emotional disadvantage, as well as exclusion from access to employment, insurance and healthcare (16).

### Employment and insurance discrimination

One debate raised concern about the potential access and use by employers of this source of health information as a selection criteria in the context of recruitment and workplace relations. Genetically based employment discrimination is the term coined to describe “the denial of workplace rights, privileges, or opportunities on the basis of information obtained from genetically based diagnostic and prognostic tests” (17). Fears of discrimination have realized in the United States where nearly two-thirds of the 1000 respondents in a survey conducted by the National Center for Genome Resources indicated they would refuse genetic testing if results were made available to employers and insurance companies, and 85% objected to employers accessing this information (18). In the Australian context, a report prepared in May 2003, and recently modified in 2012, by the joint efforts of the Australian Law Reform Commission (ALRC) and the Australian Health Ethics Committee (AHEC) of the National Health and Medical Research Council (NHMRC) offered guidelines about how to deal with the ethical, legal, and social implications of genetic information in Australia (19). The report contains specific regulations pertaining to the legalities of genetic determinism and use of genetic testing and information in employment.

The prevalence of actual cases (reported and unreported) of genetic-based employment discrimination in Australia remains largely undefined. However, several cases were identified in a 2001 study of genetic discrimination in Australia. Case were documented of employer-requested pre-employment genetic testing, as well as alleged discrimination against asymptomatic employes on the basis of information obtained from genetic test results or family history (20). One of the cases involving request of pre-employment genetic information pertained to that of a young woman with a relevant family history, who was allegedly informed that a successful job application in the public service would be conditional upon negative genetic testing for familial adenomatous polyposis. Further cases of alleged genetic-based employment discrimination were identified against existing employes, with cases involving termination of employment or demotion of individuals with positive genetic tests for familial early-onset alzheimer’s or Huntington’s disease (20).

### Emotional discrimination

Conflicting results have emerged from the limited research evaluating concerns over emotional disadvantage and genetic testing behavior. In some studies individuals reported heightened levels of disease-related anxiety up to 18 months following receipt of the test results for a hereditary cardiac condition (21) while other studies reported only temporary mild increases in general anxiety or depression (22, 23). The inconsistencies may be attributable to complexities in applying standardized testing for emotional responses. For example, the adverse emotional responses reported by individuals who underwent testing for a hereditary colorectal cancer included feelings of guilt and injustice, fear of cancer whenever a new symptom arose, use of emotion regulation strategies such as denial and immersion into work activities, difficulties communicating news to relatives, and uncertainty and worries about the future (24). These emotional concerns are difficult to translate using standard indices of distress and adjustment.

Interestingly, one study considered the impact of pre-existing psychological vulnerability and reported evidence that the highest levels of depressive symptoms occurred in participants with a high baseline stress who subsequently declined test uptake (25).

### Prior test behavior

Uptake of the hypothetical genetic cancer test correlated with a positive decision to uptake genetic testing for Down Syndrome in the immediate preceding pregnancy. This might be accounted for by consistency in the behavioral factors influencing a person to uptake a test, and reflect internal behavioral paradigms or the decision-maker’s values and beliefs. Inherently the test scenario is different. One is a test on the unborn child and the other a test of the proband themselves. The common link is the genetic nature of the test. However, previous studies have identified a key constituent in the decision-making process – the influence of the decision-makers’ values and beliefs (26–29). This could account for the correlation between the decision to uptake an initial antenatal screening test with that of a subsequent genetic screening test, since it may be postulated that the decision-maker employs similar guiding principles in the process of deciding upon test uptake.

### Prior family history

This study did not report a prior family history of genetic disease or of cancer as being significant variables associated with test uptake. There was a trend in univariate analysis for a family history of cancer to be associated with an increased test uptake (*p* = 0.07). This trend did not follow into multivariate analysis (*p* = 0.37). However, other studies have reported family history to be a factor in decision-making (2, 3).

### Strengths and weaknesses

This study has a number of strengths and weaknesses.

Strengths are the diversity of the patient population in race, religion, educational level, and cultural experience. The high recruitment rate and inclusion of non-English speaking participants are also strengths.

The study weaknesses are that recruitment came from a single institution, the inability to internally validate the responses on a second occasion and the inability to provide specific counseling about the possibility of screening and/or taking preventative measures for any cancer susceptibility.

## Conclusion

Despite the growing array of genetic tests available to identify vulnerability to certain disease states, our results suggest that when individual’s perceive there is potential for misuse of genetic information by employers upon which to base personnel decisions, or a fear of potential adverse emotional outcomes, there is reluctance to participate in genetic testing. A previous history of accepting genetic testing predisposes to testing uptake. Further systematic data is required to gage whether these concerns are founded and whether existing legislative protection is adequate.

## Conflict of Interest Statement

The authors declare that the research was conducted in the absence of any commercial or financial relationships that could be construed as a potential conflict of interest.

## References

[1] Codori AM Petersen GM Miglioretti DL Larkin EK Bushey MT Young C Attitudes toward colon cancer gene testing: factors predicting test uptake. Cancer Epidemiol Biomarkers Prev (1999) 8:345–51 10207639

[2] Evans AM Love RR Meyerowitz BE Leventhal H Nerenz DR Factors associated with active participation in a cancer prevention clinic. Prev Med (1985) 14:358–71 10.1016/0091-7435(85)90062-3 4059188

[3] Bostick RM Sprafka JM Virnig BA Potter JD Predictors of cancer prevention attitudes and participation in cancer screening examinations. Prev Med (1994) 23:816–26 10.1006/pmed.1994.1139 7855115

[4] Friedman LC Webb JA Bruce S Weinberg AD Cooper HP Skin cancer prevention and early detection intentions and behavior. Am J Prev Med (1995) 11:59–65 7748588

[5] Lloyd S Watson M Waites B Meyer L Eeles R Ebbs S Familial breast cancer: a controlled study of risk perception, psychological morbidity and health beliefs in women attending for genetic counseling. Br J Cancer (1996) 74:482–7 10.1038/bjc.1996.387 8695370 PMC2074635

[6] Chilaka VN Konje JC Stewart CR Narayan H Taylor DJ Knowledge of Down syndrome in pregnant women from different ethnic groups. Prenat Diagn (2001) 21:159–64 10.1002/1097-0223(200103)21:3<159::AID-PD20>3.0.CO;2-V 11260600

[7] Esplen MJ Madlensky L Aronson M Rothenmund H Gallinger S Butler K Colorectal cancer survivors undergoing genetic testing for hereditary nonpolyposis colorectal cancer: motivational factors and psychosocial functioning. Clin Genet (2007) 72:394–401 10.1111/j.1399-0004.2007.00893.x 17892499

[8] Godard B Pratte A Dumont M Simard-Lebrun A Simard J Factors associated with an individual’s decision to withdraw from genetic testing for breast and ovarian cancer susceptibility: implications for counseling. Genet Test (2007) 11:45–54 10.1089/gte.2006.9998 17394392

[9] Oster E Dorsey ER Bausch J Shinaman A Kayson E Oakes D Fear of health insurance loss among individuals at risk for Huntington disease. Am J Med Genet (2008) 146A:2070–7 10.1002/ajmg.a.32422 18627059 PMC2574833

[10] Suriadi C Jovanovska M Quinlivan JA Factors affecting mother’s knowledge of genetic screening. Aust N Z J Obstet Gynaecol (2004) 44:30–4 10.1111/j.1479-828X.2004.00171.x 15089865

[11] McLaughin G SMOG grading – a new readability formula. J Read (1969) 12:639–646

[12] Riley M Halliday JL Victorian Perinatal Data Collection Unit, Consultative Council on Obstetric and Paediatric Mortality and Morbidity. Births in Victoria 1999-2000. Melbourne, VIC: Perinatal Data Collection Unit Public Health Dept. of Human Services (2001).

[13] Australian Bureau of Statistics Year Book Australia. Canberra: Australian Bureau of Statistics (2001). 164 p.

[14] Smith LB Sapers B Reus VI Freimer NB Attitudes towards bipolar disorder and predictive genetic testing among patients and providers. J Med Genet (1996) 33:544–9 10.1136/jmg.33.7.544 8818938 PMC1050660

[15] Long KA Thomas SB Grubs RE Gettig EA Krishnamurti L Attitudes and beliefs of African-Americans toward genetics, genetic testing, and sickle cell disease education and awareness. J Genet Couns (2011) 20:572–92 10.1007/s10897-011-9388-3 21748660

[16] Joly Y Feze IN Simard J Genetic discrimination and life insurance: a systematic review of the evidence. BMC Med (2013) 11:25 10.1186/1741-7015-11-25 23369270 PMC3606414

[17] Billings PR Kohn MA de Cuevas M Beckwith J Alper JS Natowicz MR Discrimination as a consequence of genetic testing. Am J Hum Genet (1992) 50(3):476–82 1539589 PMC1684266

[18] Miller PS Genetic discrimination in the workplace. J Law Med Ethics (1998) 26:189–97 10.1111/j.1748-720X.1998.tb01419.x 11066876

[19] Australian Law Reform Commission and the Australian Health Ethics Committee of the National Health and Medical Research Council Essentially Yours: The Protection of Human Genetic Information in Australia (ALRC Report 960). Canberra: Australian Law Reform Commission and the Australian Health Ethics Committee of the National Health and Medical Research Council (2003).

[20] Barlow-Stewart K Keays D Genetic discrimination in Australia. J Law Med (2001) 8:250–62 12242882

[21] Hendriks KS Hendriks MM Birnie E Grosfeld FJ Wilde AA van denBout J Familial disease with a risk of sudden death: a longitudinal study of the psychological consequences of predictive testing for long QT syndrome. Heart Rhythm (2008) 5:719–724 10.1016/j.hrthm.2008.01.032 18452877

[22] Heshka JT Palleschi C Howley H Wilson B Wells PS A systematic review of perceived risk, psychological and behavioral impacts of genetic testing. Genet Med (2008) 10:19–32 10.1097/GIM.0b013e31815f524f 18197053

[23] Collins VR Meiser B Ukoumunne OC Gaff C St John DJ Halliday JL The impact of predictive genetic testing for hereditary nonpolyposis colorectal cancer: three years after testing. Genet Med (2007) 9:290–7 10.1097/GIM.0b013e31804b45db 17505206

[24] Carlsson C Nilbert M Living with hereditary nonpolyposis colorectal cancer: experiences from and impact of genetic testing. J Genet Couns (2007) 16:811–20 10.1007/s10897-007-9117-0 17705030

[25] Lerman C Hughes C Lemon SJ Main D Snyder C Durham C What you don’t know can hurt you: adverse psychologic effects in members of BRCA1-linked and BRCA2-linked families who decline genetic testing. J Clin Oncol (1998) 16:1650–4 9586874 10.1200/JCO.1998.16.5.1650

[26] van den Berg M Timmermans DRM ten Kate LP van Vugt JMG van der Wal G Are pregnant women making informed choices about prenatal screening? Genet Med (2005) 7(5):332–8 10.1097/01.GIM.0000162876.65555.AB 15915085

[27] Rowe H Fisher J Quinlivan J Women who are well informed about prenatal genetic screening delay emotional attachment to their fetus. J Psychosom Obstet Gynaecol (2009) 30(1):34–41 10.1080/01674820802292130 19308781

[28] Wynter KH Rowe HJ Fisher JR Lee M Quinlivan JA Are adolescent’ decisions about prenatal screening for Down Syndrome informed? A controlled prospective study. J Pediatr Adolesc Gynecol (2011) 24(1):29–34 10.1016/j.jpag.2010.06.006 20709585

[29] Cameron LD Muller C Psychosocial aspects of genetic testing. Curr Opin Psychiatry (2009) 22:218–23 10.1097/YCO.0b013e3283252d80 19553879

